# The therapeutic potential of sialylated Fc domains of human IgG

**DOI:** 10.1080/19420862.2021.1953220

**Published:** 2021-07-21

**Authors:** Richard J. Pleass

**Affiliations:** Department of Tropical Disease Biology, Tropical Disease Biology, Liverpool School of Tropical Medicine, Liverpool, UK

**Keywords:** sialic acid, IgG, Fc, Siglec, influenza, virus

## Abstract

Pathogens frequently use multivalent binding to sialic acid to infect cells or to modulate immunity through interactions with human sialic acid-binding immunoglobulin-type lectins (Siglecs). Molecules that interfere with these interactions could be of interest as diagnostics, anti-infectives or as immune modulators. This review describes the development of molecular scaffolds based on the crystallizable fragment (Fc) region of immunoglobulin (Ig) G that deliver high-avidity binding to innate immune receptors, including sialic acid-dependent receptors. The ways in which the sialylated Fc may be engineered as immune modulators that mimic the anti-inflammatory properties of intravenous polyclonal Ig or as blockers of sialic-acid-dependent infectivity by viruses are also discussed.

## Introduction

**Figure 1.**
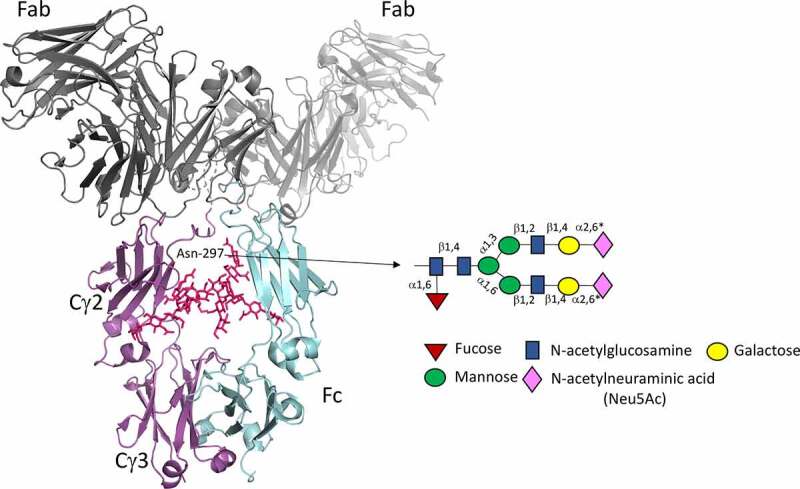
The crystal structure of human IgG1

Sialic acid-containing molecules play important roles in many fundamental immunological and pathological processes via carbohydrate–protein interactions that occur during the development of the immune system and during immune responses to pathogens.^1,2^ Examples include the regulation of fluid-phase innate immunity and modulation of leukocyte trafficking via sialylated selectin ligands.^3^ Two members of the sialic acid family (Neu5Ac and Neu5Gc) commonly occur as the terminal constituents of carbohydrate chains and are attached through post-translational modifications to glycoproteins at N- and O-linked attachment sites within the protein (Figure 1).

X-ray crystal-structure coordinates were taken from the Protein Data Bank entry 1HZH.^4^ One heavy chain is shown in cyan, the other in magenta, and the N-linked oligosaccharides attached to Asn-297 in IgG1 are shown in pink. A simplified view of the relevant N-linked sugars that may be found at Asn-297 is shown in cartoon format with the terminal N-acetylneuraminic acid (Neu5Ac) shown as pink diamonds. The Fab arms are shown in gray. *Although terminal sialylation on complex N-glycans can happen via either an α2,3-linkage or α2,6-linkage as depicted in the figure, sialic acids on human serum IgG Fc are almost exclusively α2,6-linked. Recombinant IgG or Fc expressed in Chinese hamster ovary (CHO) cells, however, have sialic acids attached through α2,3-linkages because of the lack of the α2,6-sialyltransferase gene.

Immunoglobulin G (IgG) molecules, which are critical components of the immune system, have structures and functions that can be radically affected by changes to the glycan backbone (Figure 1). Approximately 30–50 different glycan structures, with and without sialic acid, can be attached to N-linked attachment sites in IgG specified by the asparagine-X-threonine/serine (Asn-X-Thr/Ser) sequence, where X can be any amino acid other than proline.^5^ Within IgG, one such conserved site at Asn-297 in the Fc is always occupied with a glycan, while 15–20% of variably occupied N-linked sites can also arise spontaneously in the antigen-binding fragments (Fabs) during antibody development (Figure 1).^6^

The oligosaccharides attached to Asn-297 are essential for the binding and activation of FcRs and complement component C1q as this functionality is abrogated or severely curtailed by enzymatic removal or site-directed mutagenesis of the Asn-X-Thr/Ser attachment site.^7–9^ Numerous IgG-Fc crystal structures show Asn-297 glycans to be integral to the Fc structure, where they are buried within the internal cavity enclosed by the two CH2 domains (Figure 1). This buried location makes it extremely difficult for the glycans to interact directly with glycan receptors but allows them to modify the overall conformation of the Fc peptide backbone,^10^ thereby affecting the interactions with FcRs and complement component C1q (Figure 1).

More detailed studies into the types of sugars involved in this functionality have shown enhanced FcRIIIA binding and antibody-dependent cell-mediated cytotoxicity (ADCC) of IgG1 in the absence of fucose;^11,12^ enhanced FcRIIIA binding but rapid clearance from the circulation of IgG1 enriched for oligomannose structures;^13–15^ and improved solubility, anti-inﬂammatory activity, thermal stability, and circulatory half-life of terminally sialylated glycans from IgG1.^16–20^ The sialylation of IgG Fc domains also impairs complement-dependent cytotoxicity.^21^ The presence of Asn-297 terminal capping sialic acid may also prevent clearance of IgG by the asialoglycoprotein receptor that binds to terminal galactose residues of N-glycans.^22^ Therefore, glycosylation, including terminal sialylation, is important for antibody function. Consequently, this review will explore how IgG, and in particular the Fc, may be engineered to enhance interactions with sialic acid-binding receptors that play a crucial role in the anti-inflammatory properties of IgG or as therapeutic blockers of sialic-acid dependent infectivity by viruses.

### Sialic acid and the anti-inflammatory properties of IgG

Intravenously administered IgG (IVIG) is a highly successful biologic approved for treating several autoimmune diseases (ADs), including idiopathic thrombocytopenic purpura (ITP), chronic inflammatory demyelinating polyneuropathy, myasthenia gravis, and other neurological illnesses.^23^ As ~70% of the global supply (worth ~$8.9 billion in 2017) of IVIG is now used to treat ADs, it can be unavailable to other patients who desperately need it, in particular individuals with primary immune deficiency where IVIG is used as replacement therapy.^24^ Worldwide consumption of IVIG has increased over 400-fold since 1980 and currently >100 tons are consumed per annum. Global supplies of IVIG are critically limited, meaning that patients with an urgent need for the drug can have restricted access to it (https://www.alliedmarketresearch.com/intravenous-immunoglobulin-IVIG-market).

The therapeutic utility of IVIG has substantial limitations, including dependence on human donors for its manufacture, and from the fact that less than 5% of injected IVIG is therapeutically active, leading to a requirement for high doses (1–2 g/kg) when used in treating most ADs. Consequently, IVIG is expensive and adverse events due to excessive protein loading are not uncommon.^23,24^ There is thus an urgent clinical need to develop cheaper, safer, and more effective alternatives to IVIG that are effective at lower dosages, although efforts have been hindered by a lack of understanding of its likely pleiotropic mechanisms of action.^25^

Many therapeutic modes of action have been attributed to IVIG. It is beyond the scope of this review to cover all such mechanisms in detail, but several excellent review articles on the topic are available.^26–29^ Here, the focus is on the mechanisms in which sialic acid engineering approaches have been undertaken.

Changes in antibody sialylation have been associated with the evolution of autoimmune and inflammatory diseases, including rheumatoid arthritis and juvenile idiopathic arthritis, which are associated with decreased levels of IgG sialylation, and particularly of the pathogenic IgG found in inflamed joints of patients.^30,31^ Levels of sialylated IgG increase during pregnancy and correlate with remission from arthritis in pregnant women.^32^ Furthermore, pathogenic anti-proteinase three autoantibodies are less sialylated in patients with active Wegener’s vasculitis.^33^ The observation that increased levels of endogenous IgG sialylation increased the likelihood of successful treatment of Kawasaki disease in patients treated with IVIG, and that patients with severe Guillain-Barré syndrome also show lower levels of IgG sialylation despite IVIG treatment have stimulated research into converting such observations into therapies.^34,35^ These clinical observations have been substantiated in multiple mouse models of arthritis and other ADs where approximately 10-fold improvements in the severity of disease are seen with sialic-acid enriched IVIG.^20,35^ Approximately 30-fold improvements over IVIG in anti-inflammatory activity can also be demonstrated using sialylated Fc, recombinant antibodies or hypersialylated IVIG preparations (e.g., M254).^19,20,36,37^ Developed by Momenta Pharmaceuticals, Inc., M254 was shown to be well tolerated in healthy subjects,^38^ and is now being investigated in patients with ITP (Phase 1/2 study NCT03866577).

### Sialic acid engineering approaches to the Fc glycans

The controlling influence of Asn-297 oligosaccharides on Fc-mediated effector functions of antibodies has driven experimental approaches to modify them, either through glycoengineering/chemoenzymatic means,^19,39^ by mutagenesis programs on the Fc protein backbone that disrupt the protein-Asn-297-carbohydrate interface,^40^ or by expression in glycosidase-adapted transgenic cell lines.^41^ For example, the marketed humanized antibody mogamulizumab, used to treat lymphoma, is manufactured in Chinese hamster ovary (CHO) cells in which the α(1-6)-fucosyltransferase (FUT8) gene is removed, resulting in an afucosylated IgG1 with enhanced FcRIIIA-dependent tumor cell killing by ADCC.^42^ Although similar approaches have yielded enhanced sialylation of IgG, with zero to moderate improvements in binding to FcRs,^19,40,43,44^ these have not led to signiﬁcant enhancements in binding to inhibitory Siglecs that are important in controlling unwanted inﬂammation,^44–46^ a ﬁnding that others have attributed to the buried location of the Asn-297-attached glycan within the Fc.^10,47^

**Figure 2.**
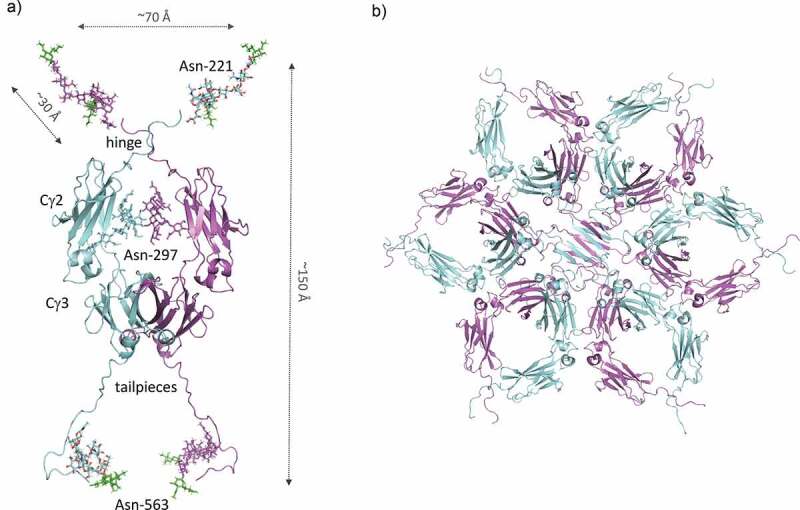
A model of sialylated Fc

Approaches to enhance the sialylation of IgG have to date focussed on modifications to the known preexisting N-linked glycosylation sites.^19,40,48,49^ My colleagues and I took an alternative approach to enhancing the sialic acid content of the Fc of IgG1 by adding the 18 amino acid tailpiece from IgM that contains an N-linked glycosylation site at Asn-563 to the C-terminus of the IgG1 Fc,^50–53^ and into which a cysteine-to-alanine substitution at Cys-575 may be introduced to prevent covalent multimerization (Figure 2). A further N-linked glycosylation site can also be introduced, if desired, to the N terminus at position Asn-221.^50^ By inserting or removing in different combinations the Asn-221, Asn-297 and Asn-563 glycosylation sites, a panel of variably glycosylated Fc monomers can be generated.^51^ As a result, tetravalent, octavalent, and dodecavalent Fc monomers can be made with respect to the attached terminal sialic acid (Figure 2a). Both non-covalent or covalently bonded higher ordered multimers, e.g., with possible sialic acid valences up to 72 for the hexamers depicted in Figure 2b, can then be generated from the basic Fc unit by the addition or removal of cysteines (Cys-309/Cys-575), either alone or in combination with the tailpiece Asn-563 glycan, that radically increase the available oligosaccharide combining sites (Figure 2b). ^47,51,53^

(A) The Asn-221, Asn-297, and Asn-563 containing Fc (D221N/C309L/C575A) containing at least eight sialic acid (colored green) combining sites was modeled on the IgG1 structure (pdb 1HZH).^4^ The N-linked glycan was attached using glycan entry 8388 (http://www.glycosciences.de) in GlyProt^54^ using the following torsion angles of 180, 340, 160, 280 and 90, 180, 160, 280 for the Asn-221 and Asn-563 attached sugars, respectively. The Asn-297 glycans are superimposed from the 1HZH IgG1 structure on which the dimer co-ordinates and final model was aligned using PyMol version 1.20. (B) Multimers of the monomeric Fc unit shown in (A) (hexamer shown) can be generated through mutagenesis approaches as previously described.^47,50,52,53,55^

Hexamers of the IgG1-Fc with no additional N-glycosylation sites introduced, other than the glycan found at Asn-297, are currently in clinical development as they have been shown to block cytotoxicity and pathological changes in experimental *in vitro* and rat models of neuromyelitis optica through mechanisms that involve interference with complement activation.^56–58^ However, potential drawbacks to the clinical use of hexamers include their large size (~350 kDa) and presence of multiple disulfide bonds that may combine to limit manufacture to scale by commercially available cell lines.

We therefore created a large panel of cysteine- and N-glycan-adapted mutants from the parent hexamer, including molecules with additional N-link attachment sites at Asn-221 and Asn-563.^50–52^ Because the Asn-297 glycan is largely buried within the Fc,^10,47^ (Figure 1) the location of Asn-221 and Asn-563 at the tips of the Fc imparts alternative functional attributes to these molecules (Figure 2a). ^51^ Five combinations of glycosylation and cysteine substitution mutants that formed either monomers or multimers and possessed different binding characteristics for FcRs, C- and I-type lectins and complement components were shortlisted.^55^ As sialylation of IgG-Fc domains is believed to be important for the anti-inflammatory effects of IVIG,^35,59,60^ molecules containing simple mono-antennary sialylation and larger more complex tri- and tetra-antennary sialylation were selected for study in an *ex vivo* model of antibody-mediated demyelination of the nervous system.^55^ The relative abundance of complex sialylated structures on mutants that did not protect (e.g., D221N/C309L/N297A/C575A), compared to the paucity of sialylated structures on mutants that did protect, argues against a direct role for sialic acid in this particular model of neurological disease.^55^ This observation is supported by studies in a number of AD models that have shown the protective effects of IVIG to be largely independent of sialylation or interactions with DC-SIGN.^44,61^ However, the sialylation state of the Fc may become critical *in vivo*, especially for neurological diseases, where the influx and efflux of IgG through the blood–brain barrier has been shown to be dependent on glycan- and sialic acid-dependent mechanisms.^62,63^

Additional heterogeneity to the glycan profiles with dramatic functional consequences can occur by expressing the molecules in either CHO or human endothelial kidney (HEK) cell lines.^51^ Unlike CHO cells, HEK cells have an active α2,6-sialyltransferase. As such, CHO-derived Fcs can only be sialylated through α2,3 linkages, whereas both α2,3 and α2,6 linkages can be found on molecules expressed by HEK cells.^51^ The potential clinical and therapeutic applications of these variably glycosylated Fc molecules are discussed below.

### Modulation of Siglecs

Through their capacity to activate or inhibit immune responses, Siglecs have become attractive therapeutic targets.^64–66^ Humans possess 14 different Siglecs that bind sialic acid. Siglec-3 (also known as CD33) belongs to a group of related Siglecs that include Siglec-5,-6, −7, −8, −9, −10, −11.^64^ These inhibitory Siglecs carry immunoreceptor tyrosine-based inhibitory motifs (ITIMs) and/or ITIM-like motifs in their cytoplasmic domains that lead to signaling cascades that suppress the activity of immune cells leading to anti-inflammatory effects.^67^ Consequently, synthetic sialylated ligands, such as sialo-polymers, sialo-nanoparticles, sialylated RNAs, and sialo-liposomes, that can bind Siglecs are being developed.^68–72^ Other promising strategies for enhancing sialylation use exogenous sialyl-transferases and donors, as well as blockade of glycosphingolipid biosynthesis.^49,73^

**Figure 3.**
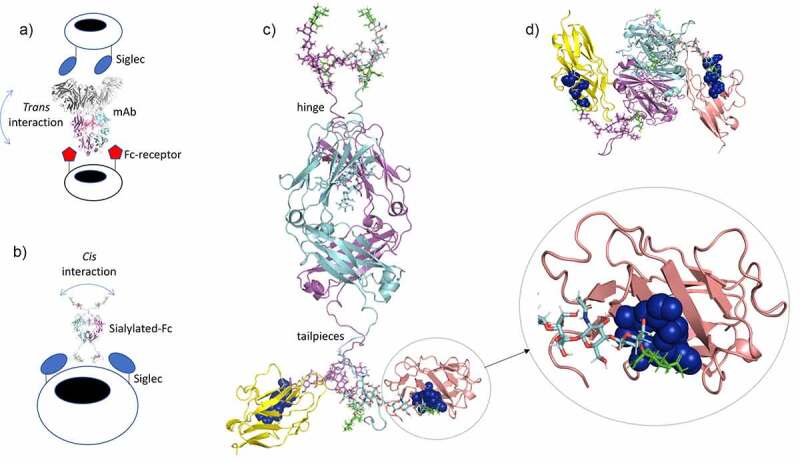
A model for the known interaction of sialylated Fc with Siglec-3 (CD33)

Many of these, including anti-CD33 monoclonal antibodies (mAbs) in clinical development, will interact with Siglecs in *trans* (Figure 3a), meaning that these synthetic ligands engage cell-surface Siglecs from the surrounding medium. Cross-linking CD33 on monocytes via antibodies induces pro-inflammatory effects, while *cis* binding of sialic acids to CD33 represses IL-1β production by monocytes.^75^ Consequently, *cis* ligands for Siglecs that reside on the same cell membrane may be superior at maintaining inhibitory signals that increase the threshold for immune activation, and this may make them useful for anti-inflammatory therapy (Figure 3b). ^67^ Indeed, the depletion of *cis* Siglec ligands has been shown to increase the activity of both macrophages and microglia, and other studies have shown that the metabolic blockade of sialic acid renders phagocytes more prone to activation.^67^ Based on these observations, it is anticipated that small high-affinity multivalent sialic acid-ligands that are better at diffusing in and around membranes than larger synthetic sialic acid-liganded molecules would have less potential for *trans*-mediated binding. Compared to other sialylated scaffolds that may bind CD33 in *trans*, e.g., antibodies, the sialylated Fc offers a number of attractive biophysical and therapeutic properties over chemically generated non-natural sialoside polymerization platforms (or even mAbs) for targeting Siglecs therapeutically in *cis* (Table 1).

**Table 1.** Different carbohydrate polymer approaches to target sialic acid-binding ligands using influenza virus as an example

	Sialylated-Fc	Sialylated-linkers^76^	mAbs
Weight (kDa)	60	1–2	150–200
Dimensions (Å)	150 x 70 x 60	30 x 30 x 30	120 x 160 x 60
Synthesis	Cell lines	Synthetic	Cell lines
Glycosylation	Native	Synthetic	Native
Sialylated (%)	80	100	0–10
Glycan attachment sites per molecule	3	3	1 for IgG1 mAbs
Multimerization potential	Yes, non-covalent or covalent modifications to the Fc possible	No, with current linkers although alternative scaffolds maybe selected	Yes, non-covalent or covalent modifications to the Fc possible
Mechanisms of action against influenza viruses	i) blocks HA ii) decoy substrate for NA iii) No ADCC & CDC	i) blocks HA ii) decoy substrate for NA iii) No ADCC or CDC	i) blocks HA ii) ADCC & CDC
IC_50_ range in HIA	nM-M	nM-M	pM-nM
Predicted renal clearance (>45 kDa cutoff)	Low	High	Negligible
Predicted half-live after *i.v*. injection *	10–15 days, Binds FcRn, high sialic acid content protects from ASGPR and MR clearance	< 1 day, No binding to FcRn or ASGPR	21 days, Binds FcRn, low sialic acid content predisposes to clearance by ASPGR and MR
Aerosol delivery	Yes	Yes	Yes
Intravenous delivery, evidence of efficacy and/or safety	Yes, Fc-fragments have been used in sick children (Debre et al., 1993) and Fc-fusions routinely used in the clinic	Unproven, majority of published scaffolds are non-human origin and potentially immunogenic (Nel et al., 2006)	Yes, mAbs routinely used in the clinic
Ease of manufacture to scale	Yes, same processes as mAbs. Easier than mAbs as no requirement for Fab domains	Unproven, requires complex chemistry	Yes, using standard mammalian cell culture e.g., in CHO-S
Cold chain dependent	Yes	Unknown	Yes
Susceptible to viral escape by mutation of pathogen	Low – carbohydrate binding essential to virus and small binding footprint of Fc	Low – carbohydrate binding essential to virus and small binding footprint of peptide	High – large binding footprint of mAb to HA epitopes prone to mutation
Differential binding to FcRs and Siglecs in cis or trans	Fine-tunable binding to FcRs and Siglecs -cis, no ADE	No binding to FcRs Broad binding to Siglecs -cis, no ADE	Binds FcRs No binding to Siglecs -trans, ADE more likely
Broader therapeutic utility	Yes, other sialic acid-dependent pathogens e.g., influenza B, SARS, MERS, adenoviruses Binds Siglecs; potential anti-inflammatory	Yes, other sialic acid-dependent pathogens e.g., influenza B, SARS, MERS, adenoviruses Unknown binding to Siglecs; potential anti-inflammatory	No, strain and epitope specific No binding to Siglecs

ADCC, antibody-dependent cell-mediated cytotoxicity; ADE, antibody-dependent enhancement; ASPGR, asialoglycoprotein receptor; CDC, complement-mediated cytotoxicity; FcRn, neonatal Fc-receptor; HA, hemagglutinin; HIA, hemagglutinin inhibition assay; i.v., intravenous; kDa, kilo Dalton; mAb, monoclonal antibody; MR, mannose receptor; NA, neuraminidase; Siglec, sialic acid-binding immunoglobulin-type lectin. *As the target location, i.e., the upper respiratory tract, is easily accessible, the molecule can be applied as an aerosol as a delivery method. Thus, commonly asked pharmacokinetic delivery issues are not relevant for this application.

(A) Potential sialylated scaffolds can target Siglecs in *trans* as depicted with anti-CD33 mAbs or, (B) may cross-link Siglecs in *cis* as illustrated with sialylated Fc-fragments disabled for receptor binding to FcRs or C1q. (C) The C-terminal tailpiece glycans on the D221N/C309L/C575A structure shown in B (rotated 90°) docked into the known sialic acid-binding site of Siglec-3 (pdb, 6D49)^74^ using PyMol. One Fc heavy chain is shown in cyan, the other in magenta, and the N-linked oligosaccharides attached to the Fc are shown in their respective heavy-chain color. Two Siglec-3 N-terminal V domains are shown in yellow and salmon, respectively. The critical sialic acid-binding residues on Siglec-3 are enlarged in the circle, and discussed in the accompanying text, are shown in blue spheres interacting with the Asn-563 glycan backbone in cyan with terminal α2,3-linked sialic acids shown in green. (D) The structure shown in C viewed from underneath.

We previously identified sialylated Fc candidates that bound CD33 yet showed minimal binding to Fc-receptors and complement C1q.^52^ Siglec-3 is a validated target for acute myeloid leukemia, and versions of the receptor that cannot bind sialic acid correlate with susceptibility to Alzheimer’s disease.^77,78^ Siglec-3 is also believed to be a common determinant of SARS-CoV-2 infection and severe respiratory distress syndrome mediated by cytokine storms.^79^

The Fc glycovariant D221N/C309L/C575A (shown in Figure 3c) bound CD33 when expressed by CHO-K1 but did not bind when expressed by HEK cells,^52^ suggesting that α2,3-linked sialosides are more important to CD33 binding as these are the only linkages attached by CHO cells. This is in line with CD33 having been reported to have a preference for α2,3- over α2,6-linked sialosides with a reported weak binding affinity (EC_50_ = 2–5 mM).^80^ The corresponding mutant in which the hinge Asn-221 glycan was removed (C309L/C575A) is still capable of binding CD33 indicating that the primary CD33 interacting glycans are located in the tailpiece at Asn-563 (Figure 3c).

We therefore generated a model of D221N/C309L/C575A to which sialic acid was attached via α2,3-linkages to Asn-221 and Asn-563 (Figure 3c,d). These structures were then docked to the known sialoside binding site of CD33 (Figure 3c,d). Only one arm of the α2,3-sialylated tailpiece glycan could be accommodated within the shallow-binding pocket of the N-terminal V-set domain, with contacts made to the conserved Arg-119, Lys-126, Lys-130, and Phe-117 residues seen in the ligand-bound crystal (Figure 3c,d). Superimposition of the docked CD33 V-set domain with the other available crystal structures from Siglecs-1, 2, and 4, offered possible models to explain why this Fc glycovariant could also bind these receptors.^51,52^

We have yet to test binding of D221N/C309L/C575A, or indeed any of the other Fc mutants, to Siglecs-6, −7, −8 and −9 largely because the commercially available Siglecs are direct fusions to the Fc. In a similar vein, we were unable to test binding to human Siglec-5 because we observed significant direct binding of Fab′_2_ detecting reagents to this receptor. The Fab′_2_-mediated binding to Siglec-5 was dependent on glycans, because treatment of the Fab′_2_ detecting reagent with neuraminidase abrogated binding to Siglec-5.^50,81^ Siglec-5 may therefore be a target for Fab glycans that have also been associated with the anti-inflammatory activity of IVIG.^6^ As the described Fc mutants can contain many different sialylated structures,^51,55^ it will be important to precisely define Siglec binding to avoid potential off-target effects given the ubiquitous expression of Siglecs on many different types of cells and tissues. Greater homogeneity (leading to enhanced specificity) to the type of sialylated structures attached to Asn-221, Asn-297, or Asn-563 may be imparted using enzymatic and/or click-chemistry approaches described by others.^19,49^

The D221N/C309L/C575A glycovariant may target CD33 in *cis* (Figure 3b), as the negatively charged N-terminal hinge-located Asn-221 glycans may discourage interactions with FcRs in *trans* that are more likely to occur with mAb approaches (Figure 3a). Furthermore, the considerably smaller size of the Fc offers other advantages, including superior penetrability of, for example, the blood–brain barrier or of hard tumors, that may be more difficult to achieve with mAbs, sialo-polymers, sialo-nanoparticles, or sialo-liposomes (Table 1). Therefore, approaches that combine antibody Fc and glyco-mimetic targeting of Siglecs may offer advantages over mAb-only or glycomimetic-only strategies.

### Sialic acid receptors of viruses

Sialic acids linked to glycoproteins and gangliosides are used by many viruses as a receptor for cell entry.^82,83^ Such viruses include significant human and animal pathogens, including influenza, parainfluenza, corona, mumps, noro, rota, adeno, and DNA tumor viruses.^82^ Attachment to sialic acid is mediated through receptor-binding proteins that are exposed at the surface of non-enveloped viruses. Some of these viruses are also equipped with neuraminidase or a sialyl-*O*-acetyl-esterase, which are receptor-destroying enzymes that can promote virus release from infected cells and neutralize sialic acid-containing soluble proteins that interfere with cell surface binding of the virus.^84^

One example is the influenza virus, which assembles hundreds of hemagglutinin (HA) trimers on its surface to recognize sialic acid-galactose linkages on target tissue.^84,85^ The monovalent interaction between HA and a typical sialylated lactosamine ligand is weak (mM range), but multivalency-enhanced interactions allow firmer adhesion at low concentration.^86,87^ Molecules that bind HA with high avidity may therefore be useful as diagnostics or anti-infective medicines.^68,86,87^ Consequently, many studies have explored multivalent scaffolds to present sialic acid to HA with the aim of blocking the interaction between virus and host receptors.^86–93^ Many different scaffolds have been used to chemically attach sialic acid, including antibodies, DNA, fullerenes, graphene, polyacrylamide, quantum dots, magnets, silver and gold nanoparticles, although biocompatibility and potential toxicological liabilities of all of these remain and none have progressed to approval for use in humans.^76,94^ Controlling the spatial distribution and number of ligand-bearing units in these oligomers can also be sub-optimal and/or ill-defined, leading to reduced binding or promiscuous binding to other sialic acid receptors (Table 1). The lack of target specificity can lead to faster *in vivo* clearance rates and may also explain reported toxicities for many polymeric inhibitors.^95,96^

**Figure 4.**
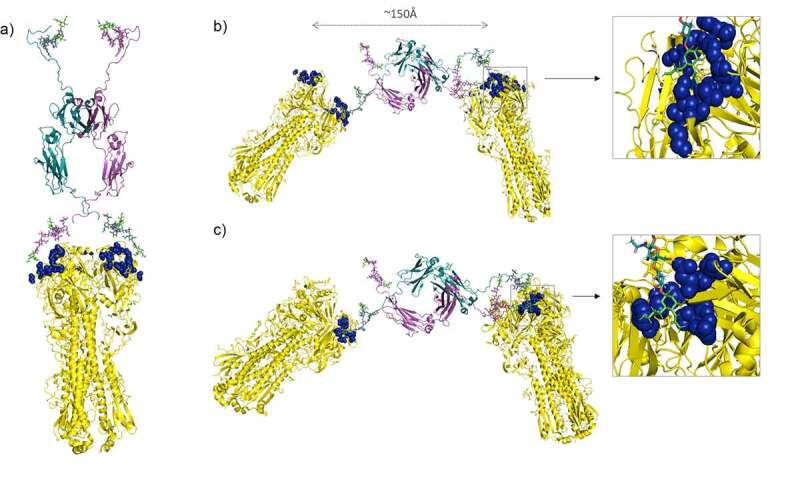
A model for the known interaction of the sialylated Fc with influenza hemagglutinin

This has driven the search for smaller, rationally designed sialylated scaffolds. Using a rigid self-assembled peptide nucleic acid complex, it has recently been shown that smaller bivalent displays of the natural sialyl-LacNAc ligand (50–68 Å between each sugar) are more effective at binding a single HA trimer and inhibiting hemagglutination by virus than longer scaffolds with sialic acid residues separated by distances >100 Å that may allow for inter-HA bridging, which may also be desirable.^86,87^ Electron micrograph analyses have shown the average distance between two adjacent HA trimers to be 101.7±0.6 Å.^86^ (Figure 4b)

(A) The N-terminal hinge domain Asn-221 attached glycans, one from each Fc (shown in magenta and cyan) docked into two of the three available sialic acid-binding sites found in the influenza A hemagglutinin trimer shown in yellow (pdb, 3LZG).^97^ Blue spheres show amino acids in the RBD of HA known to interact with sialic acid (green) and discussed in the text. (B) The location of glycans at each end of the Fc may also facilitate inter-HA binding. Close-up of the boxed binding site shown for HA from influenza A. (C) The sialylated Fc docked into the RBD from influenza B (pdb, 3BT6).^98^ Coloring of critical residues as for panels A and B.

We have previously shown that a sialylated Fc construct (molecule D221N/C309L/N297A/C575A) generated in CHO-K1 cells could potently inhibit binding to HA and blocked hemagglutination by influenza A and B viruses.^51,52^ Although D221N/C309L/N297A/C575 does not bind FcRs or C1q, other Fc mutants that partially blocked influenza B and retained FcR and C1q binding were also made, e.g., D221N/N563A/C575A.^51^ The lack of binding to FcRs or complement may be therapeutically useful, as no interference with neutralizing antibodies raised by influenza vaccines would be expected. Through additional interactions with Siglecs, the sialylated Fc can mimic the known anti-inflammatory pathways of IgG that can protect patients from overt inflammatory responses, e.g., cytokine storms that can kill.^99,100^

In contrast to CD33, binding to HA is driven by the hinge-attached glycan located at Asn-221, as its removal in the C309L/N297A/C575A mutant abrogated binding.^52^ The lower hemagglutination inhibition assay (HIA) activity seen by the C309L/N297A/C575A mutant, in which the tailpiece Asn-563 glycan is retained, was surprising as the tailpiece Asn-563 glycan can be required for the anti-influenza virus activity of human anti-HIV virus IgA.^101^ All Fc constructs tested to date in the HIA assay contain adapted human IgM tails, but the findings of Maurer et al.,^101^ suggest that different binding to HA may be acquired using the IgA backbone in which the tailpiece differs at seven of the 18 amino acids to the tailpiece found in IgM.

The narrowest and widest distances achievable between the sialic acid residues in our modeled Fc are ~30–70 Å and 110–150 Å, respectively (Figure 2a), suggesting that the sialic acid-adapted Fc may allow both intra-HA and inter-HA binding (Figure 4). The hinge and tailpiece regions of the Fc are known to be highly flexible and the attached sialic acids may therefore reach more widely spaced HA trimers on the surface of more than one virus particle.^102^ We previously observed that this octa-sialylated Fc was consistently more effective at inhibiting influenza B than influenza A viruses (EC_50_, ~30 nM vs. ~250 nM in HIA).^51^ To investigate possible structural explanations for these observations, we evaluated the molecular interactions of the D221N/C309L/N297A/C575A sialylated Fc with known crystal structures of HA from both influenza A and B viruses (Figure 4).

We docked one neuraminic acid unit from each of the two hinges into the cleft specifying the receptor-binding domain (RBD) of HA from influenza A (Figure 4a), with one face of the pyranose ring toward the bottom of the depression, and the other face exposed to solution as in published structures.^97,103,104^ All the relevant ring substituents of α-NeuAc can interact with known key residues including, Trp-153, Asp-190, Thr-136, Lys-155, Lys-221, and Glu-226 within the RBD (Figure 4a). In this model, adjacent N-acetylgalactosamine and N-acetylglucosamine can also form independent interactions with the peptide backbone of HA (Figure 4b).

Influenza B diverged from influenza A approximately 4,000 years ago^105^ and we were interested in understanding how the octa-sialylated Fc inhibited hemagglutination by the B virus more effectively than the A virus. The influenza B RBD in the globular head of HA is noticeably longer and wider, allowing the sialic acid on the Fc to be more comfortably accommodated within the RBD.^98^ (Figure 4c)

Sialic acid is also the substrate for influenza neuraminidase (NA). We do not yet know if sialylated Fcs are susceptible to cleavage by influenza NA. Although a decoy for NA may be a therapeutically attractive strategy,^106^ we have not observed a direct decay in the HIA after prolonged incubation. This suggests that the high speciﬁc avidity of these molecules for HA may reduce their susceptibility to NA, a hypothesis that ﬁts with the relatively low efﬁciency of NA (*k*_cat_ = 30–155^s−1^), together with the asymmetric distribution of NA in relation to HA on the surface of ﬁlamentous inﬂuenza viruses.^76,84,107^

To be useful, in compounds when administered intranasally or as an aerosol, the sialylated Fc needs to outcompete the sialylated mucins that viruses use, through ratchet-like interactions with HA and NA, to migrate to the underlying respiratory epithelium.^84^ Of the 15 known human mucins in the human lung, only MUC5 has been shown to give protection from inﬂuenza.^108,109^ Most sialic acid found on human mucins are O-glycosylated, and where N-linked attachments do occur, these are mostly sialylated via α2,6-linkages.^109^ Thus, we were surprised that none of the Fc leads inhibited inﬂuenza A or inﬂuenza B agglutination of human O+ erythrocytes when manufactured by HEK cells, which attach the more human type α2,6-linked sialic acid.^51^ The apparent importance of α2,3-linked N-glycans to the inhibition of both inﬂuenza A and B by the CHO-K1 Fc mutants indicates that viruses can evolve away from inhibition by mucus, whose predominant O-linked glycans are mostly α2,6-linked.^109,110^ Our working hypothesis is that HEK-expressed sialylated Fc may inhibit influenza viruses that circulate in human populations or that are propagated in cell lines that attach more human-like α2,6-linked sialic acid. The findings suggest that sialylated Fcs may also be useful blockers of coronavirus S glycoproteins that mediate attachment to oligosaccharide receptors, such as MERS-CoV, which also binds α2,3-linked, and to a lesser extent, α2,6-linked sialic acids.^82,111^

Recent reports have demonstrated that the current H3N2 viruses no longer have a strict specificity toward human-type receptors, which may result in loss of binding by the sialylated Fc to these viruses.^112^ It has become clear that H3N2 viruses maintain human-type specificity but have evolved a preference for a subset of sialylated receptors with branched glycans and extended poly-N-acetyl-lactosamine (poly-LacNAc) chains^113^ that are not attached to any of the inhibitory Fcs expressed in either CHO or HEK cells.^51^ Because this specificity is also shared with the 2009 pandemic H1N1, enhanced Fc blockers may potentially be created by manufacturing these molecules in human respiratory cell lines where such sialic acid attachments are known to occur.^114^ Alternatively, enzymatic and/or click-chemistry approaches may be used to build the required glycan structure onto the Asn-221 and Asn-563 acceptor sites as documented previously for IgG.^19,49^

In summary, multivalent sialylated IgG Fcs offer many advantages over existing approaches to deliver high-avidity blocking or triggering of sialic acid-dependent receptors, such as Siglecs (Table 1). The proven abilities of the Fc to be intravenously injected^16^ and of Fc-fusions to be delivered directly into the eye^115^ or as an aerosol to the respiratory tract^116^ are particularly noteworthy. As the binding epitope for all hemagglutinins is sialic acid and is determined by the host, these ligands are less prone to viral escape by genetic drift compared to mAbs and, unlike mAbs, are more readily manufactured and improvable through click-chemistry approaches to the glycan backbone as a consequence of introducing additional N-linked glycosylation at exposed sites in the Fc.

## Abbreviations

ADE, antibody-dependent enhancement; C1q, complement component 1q; CD, cluster designation; CH2, constant heavy 2; CHO, Chinese Hamster Ovary; Fc, fragment crystallizable; HA, hemagglutinin; HEK, human endothelial kidney; HIA, hemagglutination inhibition assay; IgG, immunoglobulin G; ITIM, Immunoreceptor tyrosine-based inhibitory motif; IVIG, intravenous immunoglobulin; Neu5Ac, N-acetylneuraminic acid; Neu5Gc, N-glycolylneuraminic acid; Siglec, sialic acid-binding immunoglobulin-type lectin.
